# The Role of Artificial Intelligence in Echocardiography

**DOI:** 10.3390/jimaging9020050

**Published:** 2023-02-20

**Authors:** Timothy Barry, Juan Maria Farina, Chieh-Ju Chao, Chadi Ayoub, Jiwoong Jeong, Bhavik N. Patel, Imon Banerjee, Reza Arsanjani

**Affiliations:** 1 Department of Cardiovascular Diseases, Mayo Clinic Arizona, Scottsdale, AZ 85054, USA; 2 Department of Cardiovascular Diseases, Mayo Clinic Rochester, Rochester, MN 55902, USA; 3 School of Computing and Augmented Intelligence, Arizona State University, Phoenix, AZ 85004, USA; 4 Department of Radiology, Mayo Clinic Arizona, Scottsdale, AZ 85054, USA

**Keywords:** echocardiography, machine learning, artificial intelligence

## Abstract

Echocardiography is an integral part of the diagnosis and management of cardiovascular disease. The use and application of artificial intelligence (AI) is a rapidly expanding field in medicine to improve consistency and reduce interobserver variability. AI can be successfully applied to echocardiography in addressing variance during image acquisition and interpretation. Furthermore, AI and machine learning can aid in the diagnosis and management of cardiovascular disease. In the realm of echocardiography, accurate interpretation is largely dependent on the subjective knowledge of the operator. Echocardiography is burdened by the high dependence on the level of experience of the operator, to a greater extent than other imaging modalities like computed tomography, nuclear imaging, and magnetic resonance imaging. AI technologies offer new opportunities for echocardiography to produce accurate, automated, and more consistent interpretations. This review discusses machine learning as a subfield within AI in relation to image interpretation and how machine learning can improve the diagnostic performance of echocardiography. This review also explores the published literature outlining the value of AI and its potential to improve patient care.

## 1. Introduction

Echocardiography is essential for the diagnosis and management of cardiac pathology. Echocardiography is one of the only imaging modalities that allows for real-time imaging and can detect various abnormalities. It is vital to accurately assess cardiac structure and function in order to assist with clinical diagnosis and guide the best treatment options for patients [1]. Despite the plethora of guidelines available for interpretation and assessment, the quality of 2D echocardiography can be challenging and susceptible to a significant degree of interobserver variability in its interpretation. The interpretation of echocardiograms remains, in part, subjectively contingent on the experience of the echocardiographer [2].

**Figure 1:**
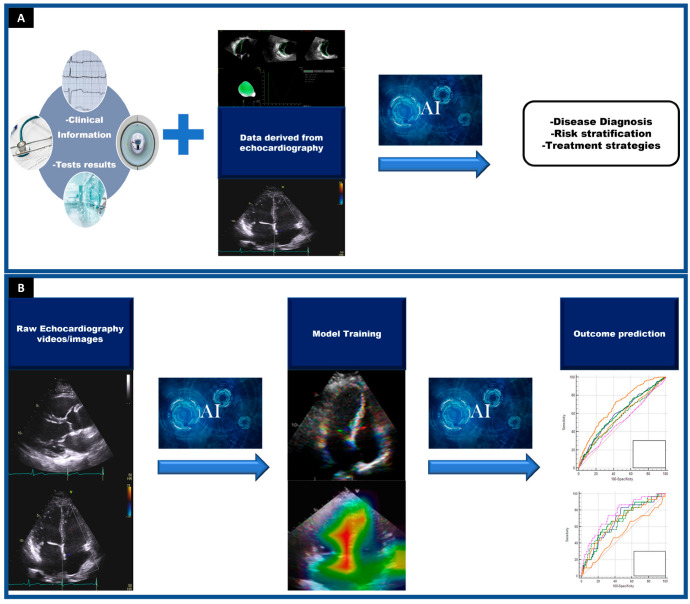
Different applications of artificial intelligence (AI) in echocardiography. **Panel** (**A**). One of the main advantages of using echocardiography in machine learning models is that these algorithms can combine data derived from echocardiography with clinical information and/or other test results to develop predictive tools with high accuracy to enhance diagnosis, risk stratification, and therapeutic strategies. **Panel** (**B**). Artificial intelligence can use raw echocardiography images/videos to automatically provide structural or functional measurements but also to identify disease states. This ability is based on AI’s capacity to automatically analyze features from images that may be too subtle to be detected by the human eye. Following training, the machine learning algorithm should be able to recognize cardiac structural and functional patterns or specific diseases. (ROC) curves are usually used to show how well the risk prediction models discriminate between patients with and without a condition.

While artificial intelligence has been around since the 1950s, in recent years there has been a strong focus on the application of AI with respect to diagnostic imaging. Machine learning and other AI techniques can recognize a variety of patterns within the imaging modalities, particularly echocardiography [3]. Echocardiography involves moving frames and can be more challenging to train AI than still images, such as those from Computed tomography (CT) or Magnetic resonance imaging (MRI) sequences. The benefit of machine learning models is that they can account for each pixel and its relationship with other pixels, as well as the associated clinical metadata. Machine learning models can be trained so that they learn what features are unique within an echocardiogram itself (Figure 1). Additionally, this can allow the models to identify images and quantify areas of interest or associations with a specific disease pattern. Combining clinician interpretation with information obtained from machine learning algorithms can refine the accuracy of echocardiography. By combining these two parameters, this will reduce inter- and intra-operator variability [3]. Moreover, this can provide additional predictive information that may be too obscure for the human eye to detect. To this end, AI may also have a potential role in expanding the availability of clinical expertise (Figure 1).

## 2. Types of Machine Learning Algorithms

Machine learning is a subfield of artificial intelligence that involves the use of algorithms to learn from and make predictions or decisions based on data [4]. Machine learning algorithms can be generally categorized based on the type of feedback or supervision the algorithm receives during the learning process, including supervised, unsupervised, semi-supervised, and reinforcement learning, as detailed below (Table 1) [5,6].

Supervised learning algorithms are provided with labeled training data, including input data and the corresponding output [5]. The goal of training is to develop a function that can map the input data to the output. Examples of supervised learning tasks include regression [7,8], which involves predicting a continuous output, and classification, which involves predicting pre-defined classes [6].

Another category of machine learning is unsupervised learning, in which the algorithm is not given labeled training data [5,9,10]. Instead, it must discover the underlying structure of the data and generate corresponding outputs. Common techniques include clustering and dimensionality reduction [6,11,12]. Unsupervised learning can be used as an exploring technique to understand the relationships in a dataset, which can be especially useful in problems without proper labeling [12,13].

A third category of machine learning is semi-supervised learning, which is a hybrid of supervised and unsupervised learning [14,15,16]. In this case, the algorithm is given a mix of labeled and unlabeled data, and it must learn from both types to make predictions or decisions [16]. Semi-supervised learning can be more effective than either supervised or unsupervised learning on its own, especially when there is a large amount of unlabeled data available [15,16].

A fourth category of machine learning is reinforcement learning [17,18], in which the algorithm learns through trial and error by interacting with its environment and receiving rewards or penalties for certain actions. This type of learning is often used in robotics and control systems, where the goal is to maximize a reward signal through a series of actions [18].

Deep learning is a special type of machine learning that involves the use of neural networks with multiple layers to learn complex patterns in data [5,19,20]. It has been particularly successful in a wide range of tasks such as image recognition [20,21], speech recognition, and natural language processing [22]. Deep learning models can learn directly from raw data and do not require manual feature engineering [19], making them well-suited for tasks with complex and high-dimensional data, such as medical images (e.g., echocardiography, chest x-ray, computed tomography, etc.) [21,23,24,25].

In summary, the categories of machine learning can be distinguished based on the type of feedback the algorithm receives during the learning process. Each category has its own unique characteristics and applications and choosing the appropriate category for a given task is an important consideration in the design of a machine learning system.

**Table 1:** Types of machine learning [5,6,17,18,19,20,21,23,24,25].

Type of Machine Learning	Examples
Supervised learning	Logistic regression and random forests
Unsupervised learning	Hierarchical clustering, tensor factorization
Reinforcement learning	Robotics and control systems
Deep learning	Image recognition (echocarcardiography, chest x-ray, computed tomography).

## 3. Automated Assessment of Myocardial Function and Valvular Disease

One of the key aspects of echocardiography is the assessment and quantification of left ventricular function and size. Left ventricular function carries significant prognostic value, and as such, it is a vital component of an echocardiogram report [26]. A plethora of techniques can be utilized for assessment of left ventricular ejection fraction (LVEF) including modified Simpson’s biplane, which is one of the most frequent methods used. This requires manual tracing of the end-systolic and end-diastolic contours in the apical four- and two-chamber views [26]. These methods and techniques for tracing biplane disc summation are subject to significant variability and have poor correlation with the gold standard Cardiac MR (CMR) [3,27].

The currently available AI technology allows for automated echocardiographic measurements. It has been demonstrated that it can increase reproducibility and bridge the gap between expert readers and novice readers. It also increases efficiency and workflow in echocardiography laboratories [28].

Knackstedt et al. examined the feasibility of automated endocardial border detection in the multicenter study utilizing a vendor-independent software package. The package applied a machine learning algorithm for images (Auto LV, TomTec-Arena 1.2, TomTec Imaging Systems, Unterschleissheim, Germany) [29]. The automated technique was reproducible and comparable to manual tracings of endocardial contours with respect to the calculation of 2D ejection fraction, left ventricular (LV) volumes, and global longitudinal strain [29]. Furthermore, this correlation was preserved when the image quality was good and moderate. However, there was a slight worsening of the correlation when the image quality was poor. Comparably, the results of automated global longitudinal strain revealed good agreement and correlation [29].

Furthermore, beyond global longitudinal strain (GLS) and LV volumes, a study by Zhang et al. demonstrated that convolutional neural networks can accurately identify echocardiographic views and provide specific measurements such as LV mass and wall thickness. In this study, a convolution neural network model was developed for echocardiographic view classification. Utilizing the output from the segmentation model, chamber dimensions were calculated according to echocardiographic guidelines [30].

### 3.1. Diastolic Function

Heart failure with preserved ejection fraction (HFpEF) is a rapidly growing global health problem. Echocardiographic analysis of diastolic function can be challenging but remains of paramount importance in the diagnosis of heart failure due to the varying clinical presentations. However, there can be errors in classification when certain comorbidities exist and current guideline-based algorithms can lead to an indeterminate classification, which can hinder the diagnosis and management of these patients [31]. There is also discrepant application of the current American Society of Echocardiograhy (ASE) 2016 diastology guidelines, even amongst experienced cardiologists. Furthermore, up to a third of patients with a diagnosis of HFpEF may be classified as having normal diastolic function by echocardiography [32]. Given the advancements in artificial intelligence and the previously described use in the assessment of systolic function, AI may provide a fresh approach to diastology by helping detect diastolic dysfunction in the one-third of patients graded as normal by echocardiographic criteria or more uniformly applying guideline criteria for more consistent interpretation of diastolic parameters [33].

Pandey et al. utilized Machine Learning (ML) to create a model to assess patients with elevated filling pressures and compared their model to the ASE 2016 diastolic guidelines grading system. Their model had a higher receiver-operating characteristic (ROC) value (0.88 vs. 0.67; *p* = 0.01) compared with the ASE guideline grades in the prediction of elevated LV filling pressures [34]. The model was also able to identify a higher risk phenotype group who had a higher risk of hospitalization and who were more likely to respond to therapy with spironolactone [34].

### 3.2. Global Longitudinal Strain

Global longitudinal strain (GLS) refers to the deformation caused by each myocardial contraction. This provides additional information about the mechanics of the myocardium utilizing speckle tracking. It has clinical utility for the detection of subclinical ventricular dysfunction which may not be seen by standard two-dimensional echocardiography, with widespread use for the detection of chemotherapy-related cardiotoxicity. Additionally, the pattern of abnormality on GLS may identify cardiac pathologies such as cardiac amyloid, hypertrophic cardiomyopathy, myocardial infarction, and constriction. As a result, there has been great interest in utilizing machine learning to assess global longitudinal strain.

Satle et al. designed a machine learning model to assess GLS in 200 patients using traditional echocardiographic views and compared it to standard speckle-tracking software (EchoPac GE) [35]. The model was able to automatically identify standard apical views, time cardiac events, and measure GLS across a variety of cardiac conditions. It demonstrated minimal differences between the two methods, with an absolute difference of 1.8% [3,35]. The method utilized was rapid, taking less than 15 s per study with AI compared to 5–10 min with the conventional method [35].

## 4. The Role of AI in Identifying Disease States

**Figure 2:**
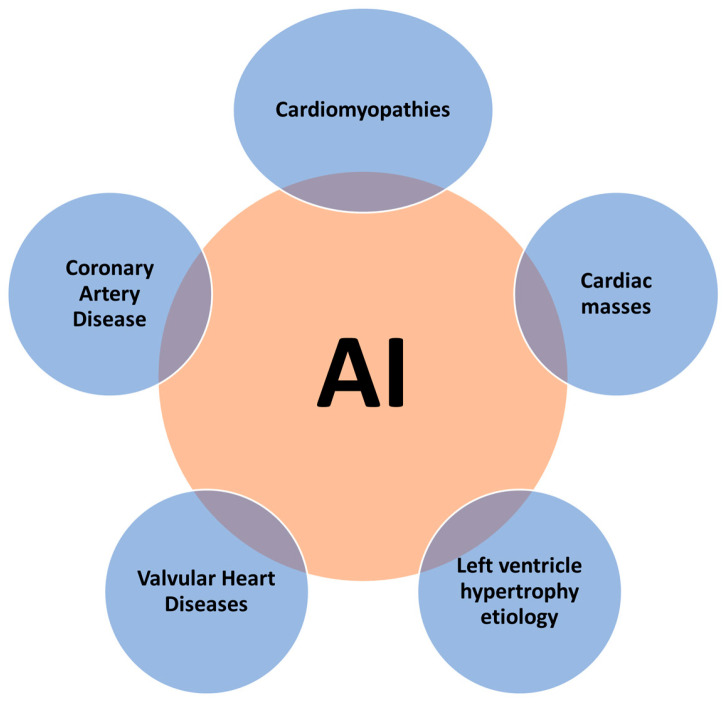
Potential clinical applications of artificial intelligence in echocardiography to identify disease states.

The rationale behind the use of AI in echocardiography to identify disease states is based on its capacity to automatically analyze features from images and data that are beyond human perception [36]. During routine echocardiography, a huge volume of potentially diagnostic information could be underutilized, considering that the totality of data generated can be hard to interpret by human experts in a short time period [37]. AI can help identify the true value of these undiscovered findings and can analyze this information faster than human experts. Therefore, the potential clinical applications of AI in echocardiography are rapidly increasing, including the identification of specific disease states and processes, such as valvular heart diseases, coronary artery disease, hypertrophic cardiomyopathy, cardiac amyloidosis, cardiomyopathies, and cardiac masses (Figure 2).

### 4.1. Valvular Heart Disease

In the field of valvular heart diseases, the focus of AI has been on the echocardiographic quantification of the severity of valve disorders and the identification of high-risk populations [36]. Using image recognition algorithms, valve disease states have been directly detected from raw images, but images have also been integrated with clinical information to identify new predictors of disease progression. Previous studies developed highly accurate algorithms based on images that could establish the severity of mitral and aortic valve disease, recognize the presence of prosthetic valves, and identify rheumatic heart disease [38,39,40]. Further progression in this field could transform how patients with valve diseases are evaluated and managed, as deep learning algorithms could simulate or replace the multimodal evaluation currently required [36].

In a recent study including almost 2000 patients with aortic stenosis, AI integrated echocardiography measurements to improve the classification of disease severity and to identify high-risk subgroups [41]. The identification of higher-risk subjects in this study (higher aortic valve calcium scores, larger late gadolinium enhancement, higher biomarker levels, and greater incidences of negative clinical outcomes) has the potential to optimize the timing of aortic valve replacements [41]. In another recent publication including a large training (*n* = 1335) and validated (*n* = 311) cohort, a framework for the automatic screening of echocardiographic videos for mitral and aortic disease was developed [42]. This deep learning algorithm was able to classify echocardiographic views, detect the presence of valve heart disease, and quantify disease severity with high accuracy (AOC > 0.88 for all left heart valve diseases) [42]. These novel findings support the effectiveness of an automated framework, trained on routine echocardiographic datasets, to screen, classify, and quantify the severity of conditions that are frequent in medical practice.

### 4.2. Coronary Artery Disease

Cardiac imaging is key for the effective management of patients with coronary artery disease [43]. However, regional wall motion abnormalities traditionally need to be subjectively identified by operators, and interobserver and intraobserver variability can be high [44]. To overcome this issue, an automated image processing pipeline was recently developed to extract geometric and kinematic features from stress echocardiograms [45]. This machine learning model obtained high classification accuracy (specificity of 92.7% and a sensitivity of 84.4%) for the identification of patients with severe coronary artery disease [45]. These results support the use of AI for the analysis of stress echocardiograms to provide automated classifications and to improve accuracy, inter-reader agreement, and reader confidence. Moreover, these findings are especially important when considering that the interpretation of stress echocardiography is widely recognized as one of the most challenging activities for echocardiographers [46].

Another potential implementation of AI in the field of coronary artery disease could be the differentiation between diseases that commonly present with signs and symptoms similar to an acute coronary syndrome. In that sense, a novel cohort study developed a real-time system for fully automated interpretation of echocardiogram videos to differentiate TakoTsubo syndrome from acute myocardial infarction [47]. While this model demonstrated to be more accurate than expert cardiologists in echocardiography-based disease classification, further studies are needed before clinical application.

Lastly, AI models could potentially provide a prediction of left ventricular recovery after coronary syndromes. One study developed a method based on the texture parameters of echocardiograms to evaluate left ventricular function recovery one year after myocardial infarction [48]. Even though the preliminary results were promising (the estimated prediction error was lower than 30%), further studies are warranted for clinical application.

### 4.3. Etiology Determination of Increased Left Ventricular Wall Thickness

In cases of increased left ventricular wall thickness, conventional echocardiography may be not sufficient for the etiological diagnoses, and more complex imaging modalities are usually needed. Myocardial texture is generally difficult to assess and quantify in routine echocardiography using only the human impression [49]. One study used echocardiography-AI-based myocardial texture analysis to differentiate hypertrophic cardiomyopathy, hypertensive heart disease, and uremic cardiomyopathy [50]. Hypertrophic cardiomyopathy showed the most homogeneous myocardial texture and was significantly different from the other diagnosis, thus supporting AI-based myocardial texture features as a potential approach to left ventricle hypertrophy etiology differentiation.

Another study investigated the diagnostic value of a machine learning framework that incorporates echocardiographic data for automated discrimination of hypertrophic cardiomyopathy from physiological hypertrophy seen in athletes [51]. This AI model showed increased sensitivity and specificity compared with conventional parameters, suggesting that the use of echocardiography images in machine learning algorithms can assist in the discrimination of physiological versus pathological patterns of hypertrophic remodeling.

Cardiac amyloidosis is characterized by left ventricular hypertrophy and can mimic hypertrophic cardiomyopathy. The impact of cardiac amyloidosis on cardiovascular imaging has been widely described, but isolated echocardiography findings have not been sufficiently specific or sensitive to be used as definitive diagnostic tools for this disease. Recently, a video-based echocardiography model for cardiac amyloidosis using only the apical four-chamber view demonstrated very good performance (C-statistics of 0.96) and outperformed expert human readers in a study including five academic medical centers across two countries [52]. Overall, the model’s superior performance was more apparent for Transthyretin Amyloidosis (ATTR) than AL amyloidosis. A second cohort study developed an AI-guided workflow that automatically quantified left ventricle wall thickness on echocardiography while also predicting the cause of left ventricle hypertrophy as either hypertrophic cardiomyopathy or cardiac amyloidosis [53]. This deep learning model accurately identified subtle changes in left ventricle wall geometric measurements and the causes of hypertrophy, thus providing a more efficient clinical evaluation of this group of patients.

In one additional study, authors developed a deep learning algorithm for the differential diagnosis of common left ventricular hypertrophy etiologies (hypertensive heart disease, hypertrophic cardiomyopathy, and AL-cardiac amyloidosis). In this research, a convolutional neural network long short-term memory algorithm was constructed to classify the three diagnoses using five standard echo views (parasternal long-axis, parasternal short-axis, apical four-chamber, apical two-chamber, and apical three-chamber). The study population included a training (*n* = 620), a validation (*n* = 155), and a test cohort (*n* = 155). In the test cohort, the Area under the curve (AUC) for the AI model was 0.962 for hypertensive heart disease, 0.982 for hypertrophic cardiomyopathy, and 0.996 for AL-cardiac amyloidosis. The overall diagnostic accuracy was significantly higher for the deep learning algorithm than for echocardiography specialists, therefore supporting that the use of AI can improve the diagnostic process in patients with left ventricular hypertrophy [54].

### 4.4. Cardiomyopathies

AI-assisted diagnosis of cardiomyopathies can be based on ventricular segmentation, measurement of volumes, and automatic assessment of myocardial function and motion [48,49]. One of the most significant benefits of AI in this field may be the potential improved diagnostic performance, particularly in the early stages of some cardiomyopathies where no obvious structural echocardiographic signs may be detected by human perception [49].

Automatic detection of dilated cardiomyopathy from echocardiography videos has been proposed by previous studies. A machine learning framework based on support vector machines was used in one study to separate normal from dilated left ventricles [55]. Even though the performance of the classification showed promising results (classification accuracy was 78%), more information is needed before considering clinical application [55].

Deep learning algorithms were developed to distinguish specific cardiomyopathies using echocardiography movies. One study used AI-assisted diagnosis to differentiate cardiac sarcoidosis from healthy subjects. The diagnostic accuracy of this AI algorithm based on echocardiography videos was not significantly different from the interpretation of the echocardiography movies by human experts [56]. A more recent study proposed a machine learning algorithm based on clinical and speckle-tracking echocardiography data to distinguish between constrictive pericarditis and restrictive cardiomyopathy [57]. The associative memory classifier used in this study showed a short learning curve, achieving over 90% of asymptotic accuracy with only 30% of the data trained, and achieved a diagnostic AOC of 89.2%, which was superior to the conventional echocardiographic variables (early diastolic mitral annular velocity and longitudinal strain).

A recent study reported an end-to-end deep learning framework that differentiates four common cardiovascular diseases (Atrial Septal Defect, Dilated Cardiomyopathy, Hypertrophic Cardiomyopathy, and prior Myocardial Infarction) from normal subjects. Interestingly, this study included 1807 echocardiographic videos obtained during standard clinical care of patients from ultrasound equipment from several different manufacturers and models, thus broadening the application of AI-assisted echocardiography in different medical settings. Moreover, the algorithm identified anatomic regions of interest relevant to each diagnosis, in a similar fashion to an echocardiographer’s approach to interpretation (interatrial septum for atrial septal defect, the left ventricular chamber for dilated cardiomyopathy, the interventricular septum for hypertrophic cardiomyopathy, and more variable patterns for prior myocardial infarction). The performance of this model was comparable to that of the consensus of three senior cardiologists. These results also demonstrate how AI-assisted echocardiographic video image analysis enhances the accuracy of disease diagnostic classification [58].

### 4.5. Intracardiac Masses

Correct echocardiographic diagnosis of the etiology of intracardiac masses can be challenging but highly important, as different treatment options are possible for diverse types of cardiac masses (thrombosis, tumors, or vegetation), and this often requires further upstream testing with advanced imaging, such as MRI, for further characterization. AI technology could be applied to classify and recognize intracardiac masses, and previous research presented classification and segmentation results of intracardiac masses in echocardiograms using texture analysis [59]. This analysis was able to reflect some physiological properties of analyzed heart tissues. A more recent study investigated whether transesophageal echocardiography assisted with a computer-aided diagnostic algorithm was superior to the conventional approach in diagnosing left atrial thrombi in patients with atrial fibrillation [60]. The AI-derived algorithm significantly improved the diagnostic accuracy for left atrium thrombi when compared with the traditional approach by experts.

## 5. Limitations

Despite the tremendous advantages of the use of AI in cardiology and medicine overall, it is not without limitations. Unquestionably, AI can analyze images efficiently and accurately, with the extra ability to save time in the diagnostic processes when compared with human experts. However, important limitations and concerns may arise. AI “black box” models are created directly from raw data by algorithms, meaning that humans, even those who design them, cannot understand how variables are being combined to make predictions. This fact implies that results of AI models are sometimes impossible to interpret and verify from a clinical point of view [61].

Most of the studies regarding the clinical applications of AI have been retrospective and AI algorithms still need to be validated in large multicenter studies [49]. Additionally, some machine learning models use labeled data, thus accepting the labels provided by scientists or by “real world” data as perfect truths, even when it is known that this approach is not free from potential bias in the labelling process. The quality of input data is critical for the development of robust AI models.

Additionally, there are significant legal and ethical issues pertaining to the use of AI in medicine. AI applications regularly require large databases and registries containing sensitive patient information, which acts as a substrate to train the AI model using machine learning algorithms [62]. This calls into question the potential security breaches which could result in large data leaks. As a result, this may leave sensitive patient information compromised. Over time, patients may feel uncomfortable when providing their data for AI applications in the event of rising security breaches, which therefore may limit future prospective trials.

Lastly, clinical applications of AI in echocardiography also face difficulties from a technical point of view. In addition to the vendor-dependent setup differences, AI clinical applications can also be affected by the frequent inability to obtain optimal image quality or accurate views [61]. In those cases, the accuracy of the models could be affected, or nonstructural/suboptimal echocardiographic data would need careful preprocessing by operators.

## 6. Conclusions

Advances in AI applications in cardiology and echocardiography are rapidly expanding, with the potential to revolutionize patient care. AI algorithms may aid in the detection, classification, diagnosis, and prognostication of cardiac abnormalities. They offer the promise of enhanced workflow efficiency, improved reproducibility, and higher diagnostic accuracy, and may represent a cost-effective tool to address the upsurge in the demand for cardiac imaging. There are many obstacles that need to be overcome to permit AI to be used in clinical practice, including the paucity of data pertaining to AI and clinical outcomes. Further research is needed in the form of prospective studies to determine their accuracy and effectiveness and how AI can affect clinical outcomes.

## Data Availability

Not applicable.
